# Prognostic Accuracy of WHO Growth Standards to Predict Mortality in a Large-Scale Nutritional Program in Niger

**DOI:** 10.1371/journal.pmed.1000039

**Published:** 2009-03-03

**Authors:** Nathanael Lapidus, Francisco J Luquero, Valérie Gaboulaud, Susan Shepherd, Rebecca F Grais

**Affiliations:** 1 Epicentre, Paris, France; 2 European Programme for Intervention Epidemiology Training (EPIET), European Centre for Disease Prevention and Control (ECDC), Stockholm, Sweden; 3 Médecins Sans Frontières, Paris, France; Institute of Child Health, United Kingdom

## Abstract

**Background:**

Important differences exist in the diagnosis of malnutrition when comparing the 2006 World Health Organization (WHO) Child Growth Standards and the 1977 National Center for Health Statistics (NCHS) reference. However, their relationship with mortality has not been studied. Here, we assessed the accuracy of the WHO standards and the NCHS reference in predicting death in a population of malnourished children in a large nutritional program in Niger.

**Methods and Findings:**

We analyzed data from 64,484 children aged 6–59 mo admitted with malnutrition (<80% weight-for-height percentage of the median [WH]% [NCHS] and/or mid-upper arm circumference [MUAC] <110 mm and/or presence of edema) in 2006 into the Médecins Sans Frontières (MSF) nutritional program in Maradi, Niger. Sensitivity and specificity of weight-for-height in terms of Z score (WHZ) and WH% for both WHO standards and NCHS reference were calculated using mortality as the gold standard. Sensitivity and specificity of MUAC were also calculated. The receiver operating characteristic (ROC) curve was traced for these cutoffs and its area under curve (AUC) estimated. In predicting mortality, WHZ (NCHS) and WH% (NCHS) showed AUC values of 0.63 (95% confidence interval [CI] 0.60–0.66) and 0.71 (CI 0.68–0.74), respectively. WHZ (WHO) and WH% (WHO) appeared to provide higher accuracy with AUC values of 0.76 (CI 0.75–0.80) and 0.77 (CI 0.75–0.80), respectively. The relationship between MUAC and mortality risk appeared to be relatively weak, with AUC = 0.63 (CI 0.60–0.67). Analyses stratified by sex and age yielded similar results.

**Conclusions:**

These results suggest that in this population of children being treated for malnutrition, WH indicators calculated using WHO standards were more accurate for predicting mortality risk than those calculated using the NCHS reference. The findings are valid for a population of already malnourished children and are not necessarily generalizable to a population of children being screened for malnutrition. Future work is needed to assess which criteria are best for admission purposes to identify children most likely to benefit from therapeutic or supplementary feeding programs.

## Abstract 2

Rebecca Grais and colleagues assess the accuracy of WHO growth standards in predicting death among malnourished children admitted to a large nutritional program in Niger.

## Editors' Summary

**Background.:**

Malnutrition causes more than a third of child deaths worldwide. The World Health Organization (WHO) estimates there are 178 million malnourished children globally, all of whom are vulnerable to disease and 20 million of whom are at risk of death. Poverty, rising food prices, food scarcity, and natural disasters all contribute significantly to malnutrition, but children's lives can be saved if aid agencies are able to identify and treat acute malnutrition early. This can be done by comparing a child's body measurements to those of healthy children.

In 1977 the US National Center for Health Statistics (NCHS) introduced child growth reference charts describing how US children grow. The charts enable the height of a child of a given age to be compared with the set of “percentile curves,” which show, for example, whether the child is on the 90th or the 10th centile—that is, whether taller than 90% or 10% of their peers. These NCHS reference charts were subsequently adopted by the WHO for international use. In 2006, the WHO began to use new growth charts, based on children from a variety of countries raised in optimal environments for healthy growth. These provide a standard for how all children *should* grow, regardless of ethnic background or wealth.

**Why Was This Study Done?:**

It is known that the WHO standards and the NCHS reference differ in how they identify malnutrition. Estimates of malnutrition are higher with the WHO standard than the NCHS reference. This affects the cost of international programs to treat malnutrition, as more children will be diagnosed and treated when the WHO standards are used. However, it is not known how the different growth measures differ in predicting which children's lives are at risk from malnutrition. The researchers saw that the data in their nutritional program could help provide this information.

**What Did the Researchers Do and Find?:**

The researchers examined data on the body measurements of over 60,000 children aged between 6 mo and 5 y enrolled in a Médecins sans Frontières (MSF) nutritional programme in Maradi, Niger during 2006. Children were assessed as having acute malnutrition (wasting) and enrolled in the feeding program if their weight-for-height was less than 80% of the NCHS average, if their mid-upper arm circumference (MUAC) was under 110 mm (for children 65–110 cm), or they had swelling in both feet.

The authors evaluated three measures to see which was most accurate at predicting that children would die under treatment: low weight-for-height as measured against each of the WHO standard and NCHS reference, and low MUAC. For each measure, they compared the proportion of correct predictions of death (sensitivity) and the proportion of correct predictions of survival (specificity) for a range of possible cutoffs (or thresholds) for diagnosis.

They found that the WHO standard gave more accurate predictions than the NCHS reference or the MUAC of which children would die under treatment. The results were similar when the children were grouped by age or sex.

**What Do these Findings Mean?:**

The results suggest that, at least in this population, the WHO standards are a more accurate predictor of death following malnutrition. This agrees with what might be expected, as the WHO standard is more up-to-date as well as aiming to show how healthy children from a range of settings should grow.

Nevertheless, an important limitation is that the children in the study had already been diagnosed as malnourished and were receiving treatment. As a result, the authors cannot say definitively which measure is better at predicting what children in the general population are acutely malnourished and would benefit most from treatment.

It should also be noted that children were predominantly entered into the feeding program by the weight-for-height indicator rather than by the MUAC. This may be a reason why the MUAC appears worse at predicting death than weight-for-height. Missing and inaccurate data, for instance on the exact ages of some children, also limit the findings.

In addition, the findings do not provide guidance on the cutoffs that should be used in deciding whether to enter a child into a feeding program. Different cutoffs represent a trade-off between treating more children needlessly in order to catch all in need, and treating fewer children and missing some in need. The study also cannot be used to advise on whether weight-for-height or the MUAC is more appropriate in a given context. In certain crisis situations, for instance, some authorities suggest it may be more practical to use the MUAC, as it requires less equipment or training.

**Additional Information.:**

Please access these Web sites via the online version of this summary at http://dx.doi.org/10.1371/journal.pmed.1000039.

- The UN Standing Committee on Nutrition homepage publishes international briefings on nutrition as a foundation for development
- The US National Center for Health Statistics provides background information on its 1977 growth charts and how they were developed in the context of explaining how they differ from revised charts produced in 2000
- The World Heath Organization publishes country profile information on its child growth standards and also on Niger
- Médecins sans Frontières also provides information on its work in Niger
- The EC-FAO Food Security Information for Action Programme is funded by the European Commission (EC) and implemented by the Food and Agriculture Organization of the United Nations (FAO). It aims to help nations formulate more effective anti-hunger policies and provides online materials, including a guide to nutritional status assessment and analysis, which includes information on the contexts in which different indicators are useful

## Introduction

In 2006, the World Health Organization (WHO) introduced the Child Growth Standards for assessing the growth and development of children from birth to 60 mo of age, in order to improve upon the 1977 National Center for Health Statistics (NCHS) international reference. Important differences have been highlighted in the diagnosis of malnutrition when comparing the WHO standards to the NCHS reference [1], their impact on measured prevalence of acute malnutrition (the use of WHO standards generally involves a significant increase in the prevalence of severe malnutrition) [2], and their operational implications (resources and program costs are consequently increased) [3]. However, the relationship of the two growth references with respect to their sensitivity and specificity as a prognostic indicator for mortality has not yet been studied. Findings in this area are important, as malnutrition remains a global public health problem and decisions about recommended indicators may have a major impact on costs and number of children included in programs worldwide.

Here, we compare the relationship between anthropometric status at admission according to each of these references and risk of death in moderately or severely malnourished children admitted to the Médecins Sans Frontières (MSF) nutritional program in Maradi, Niger. We compared the sensitivity and specificity of weight-for-height (WH), expressed as Z scores (WHZ), or percentage of the median (WH%), using WHO growth standards or NCHS reference. We also assessed the use of the mid-upper arm circumference (MUAC) as an indicator of mortality risk.

## Methods

### Study Setting

Niger, a landlocked country of the Sahel in Africa, has a population of approximately 14 million people. One of the poorest countries in the world, Niger faces numerous challenges, notably an arid climate, recurrent drought, demographic pressure, and scarcity of resources. Many people in Niger experience chronic food insecurity, marked each year with a “lean season” or “hunger gap.” During these months, the intake of food—both in terms of quality and quantity—is insufficient to meet the nutritional needs of young children. Disease, including malaria, diarrhea, and acute respiratory infections, combines with the high prevalence of malnutrition to contribute to one of the highest under-5-y mortality rates in the world [4].

In 2006, MSF operated 11 outpatient centers attached to integrated health centers and two inpatient referral feeding centers in Maradi, Niger. The prevalence of acute malnutrition in Maradi varies throughout the year because of the seasonality of the agricultural harvest and food insecurity. In 2006, the prevalence of malnutrition in Maradi was estimated to range from 6.8% (October 2006) to 11.6% (January–May 2006) for global acute malnutrition, and 0.6% (October 2006) to 1.0% (January–May 2006) for severe acute malnutrition [5,6], using < −2 and < −3 Z-score criteria respectively, according to the NCHS reference.

### Study Population

There were 68,001 children aged 6–59 mo admitted with moderate or severe acute malnutrition from January 1 to December 31, 2006 to the MSF therapeutic feeding program in Maradi. Children presenting to feeding centers were screened for malnutrition using weight, height (or recumbent length for children <2 y), MUAC, and presence of edema (kwashiorkor).

Children were eligible for program admission if they had WH <80% of the NCHS reference median and/or MUAC <110 mm (for children 65 cm–110 cm) and/or bipedal edema. Discharge occurred at WH >80% of the median after two consecutive weight measurements, or at WH >85% of the median at one weight measurement after early November.

Since 2003, the MSF nutritional program in Maradi has used an outpatient strategy [7], where children with sufficient appetite and without complications were offered home-based outpatient treatment with the provision of ready to use therapeutic food (RUTF). Outpatient treatment consists of two sachets per day of RUTF (Plumpy'nut, 500 kcal per sachet) plus vitamin A and folic acid supplementation, an antihelminthic, and measles vaccination. Each child was screened for malaria with a rapid diagnostic test and an artemisinin-based combination treatment was given to all children with positive tests. A course of amoxicillin was given to all children meeting criteria for severe acute malnutrition without complication. Acutely malnourished (severe or moderate) children with medical complications (e.g., severe malaria, severe diarrhea, severe lower respiratory tract infection, or anorexia) were managed in an inpatient setting until their condition was stabilized. Inpatient treatment initially consisted of the same systematic medical treatment (with antibiotic or antimalarial therapy adapted to the child's condition) and therapeutic F-75 diet given in small, frequent meals (eight meals per day, 100 kcal/kg/day). As their condition improved and appetite returned, children were transitioned to F-100 (200 kcal/kg/day) and then F-100 plus RUTF. Children without complications with WH between 70% and 80% of the median did not systematically receive antibiotics after April 2006.

Mothers of the children were asked to return to the outpatient centers on a weekly basis for follow-up, during which height and weight were measured and a complete medical screening was conducted to assess the presence of clinical signs including fever, dehydration, respiratory disorders, anaemia, and state of consciousness. At every visit, the following week's supply of RUTF was distributed. Trained MSF nutrition assistants carried out all anthropometric measurements on the children using standardized methods and calibrated instruments. Height (recumbent length if <85 cm) was measured to the nearest 0.1 cm using a measurement board. Weight was measured to the nearest 0.1 kg using a hanging Salter scale, and to the nearest 0.01 kg using a mechanical SECA 75 scale as of July 2006. MUAC was measured to the nearest mm using a nonstretchable tape.

### Data Collection

Information was gathered from the medical records of discharged children and entered weekly. At admission, data were collected on age, sex, height, weight, MUAC, edema status, and selected clinical signs. Height and weight were measured at week 1, week 2, and discharge. Type of discharge (recovery, death, nonresponse, and loss to follow-up) and length of stay by type of care (inpatient versus outpatient) were also collected. These variables were entered into an Epidata database (Odense, Denmark). For the analysis presented here, we used admissions data based on age, weight, height, MUAC, and presence of edema. The type of discharge was the main outcome measure (death versus any other type of discharge). Nonresponse to treatment was defined as follows: children failing to register sufficient weight gain to meet WFH >80% median on two consecutive weeks after 3 mo in the program; this in the absence of tuberculosis, HIV, or other chronic illness and after passage through the inpatient center for supervised feeding. Children classified as lost to follow-up were defined as those not presenting for treatment at an outpatient center after four consecutive visits (or three consecutive visits in the district of Guidam Roumdji). There was no active search for those children lost to follow-up.

### Data Analyses

**Figure 1:**
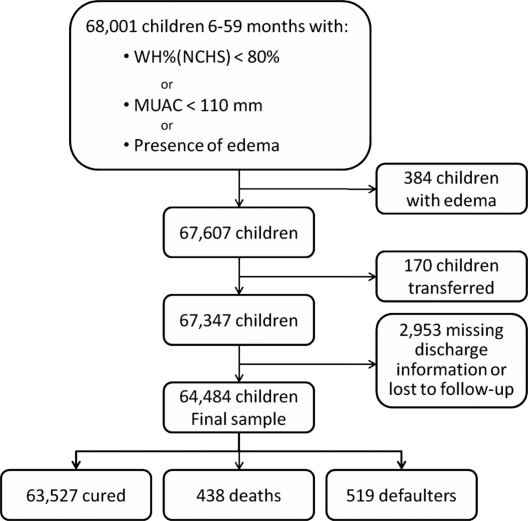
Exclusion Criteria Applied in the Study and Final Sample Size

We excluded children with documented edema at admission from the analysis as their anthropometric measures were likely to have had a different relationship with mortality in children with edema (*n =* 394). Children with missing information on edema status on admission (*n =* 10,724) were considered to not have edema, since medical staff were explicitly asked to note when edema was present. Children transferred to or from a treatment program other than the MSF program were excluded (*n =* 170), since the reason for transfer was not documented and their final status was unknown. Children lost to follow-up or with missing final status (*n =* 2,953) were excluded from the analysis (Figure 1).

We examined the following anthropometric indicators at admission in relation to death during the stay: WHZ and WH% for both NCHS reference and WHO standards and for the MUAC. We analyzed all children in the database as well as stratifying by sex and/or age (groups: 6–11 mo, 12–23 mo, and 24–59 mo).

For the NCHS reference, we calculated weight-for-length for children <85 cm and WH for children ≥85 cm, using EpiNut (Epi Info 6.0) software. For the WHO standards, we calculated weight-for-length for children <24 mo and WH for children ≥24 mo, as recommended by the WHO. We calculated WHO Z scores using the *igrowup* macro available at http://www.who.int/childgrowth/software/en/. We also calculated WHO WH% values using the published intervals of 0.5 cm for length/height between 45–120 cm with linear interpolation.

The sensitivity and specificity of these indicators in relation to mortality were studied simultaneously for any possible cutoff. The receiver operating characteristic (ROC) curve was traced for these cutoffs and its area under curve (AUC) was estimated. AUC measures the discriminating accuracy of the indicator, i.e., the ability of the indicator to correctly classify children who will or will not die during their stay [8].

All analyses were conducted with the use of R software 2.6.0 (R Development Core Team; R Foundation for Statistical Computing [http://www.R-project.org]).

### Ethical Considerations

This study used routine program monitoring data from the MSF nutritional program. This program was conducted in collaboration with the Ministry of Health of Niger via a memorandum of understanding, which is the normal and usual operating procedure for NGOs operating nutritional programs. No supplementary interventions were given to participants. All data were anonymized when entered into the database and identification numbers were coded. No ethnic or identifying information was encoded. Individual oral informed consent was obtained from the parent or caregiver at the child's admission to the program.

## Results

Data from 64,484 children were analyzed. There were more girls (55.5%) admitted to the program than boys. The average age at admission was 19.5 mo (standard deviation 8.6). The total number of deaths during care was 438. The highest proportion of deaths occurred in the youngest age group (6–11 mo) (16.8%; 95% confidence interval [CI] 14.3–19.8%) (Table 1). Most children were moderately malnourished (94.8% had a weight higher than 70% of the NCHS median) and the mean MUAC was 124 mm (3.0% with MUAC <110 mm). Table 2 displays anthropometric indicators calculated from measurements on children at admission. Only 13 children had a HIV-positive serology, and none of them died during the study period.

**Table 1:**
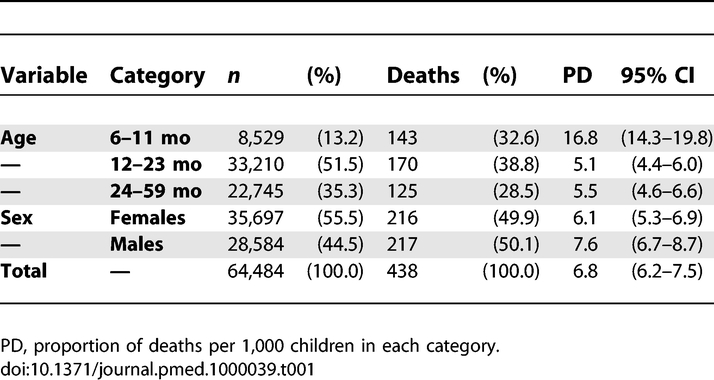
Description of the Study Population by Sex and Age: Number and Percentage of Children, Number and Percentage of Deaths, Proportion of Deaths per 1,000 Children in Each Category, and 95% CIs

**Table 2:**
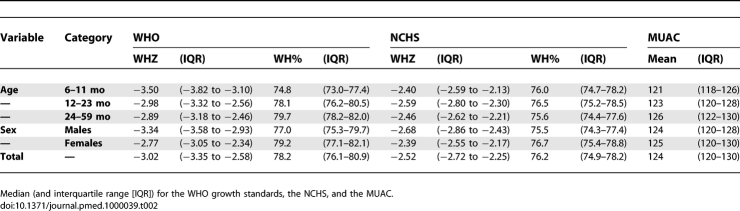
Anthropometric Indicators Calculated from Measurements on Children at Admission

### NCHS and WHO Comparison

**Figure 2:**
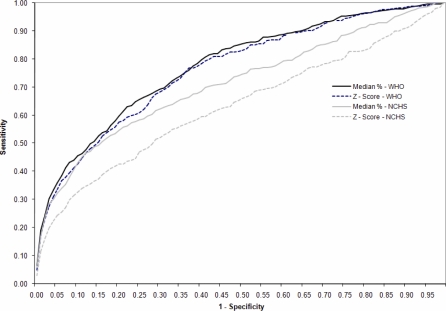
ROC Curves for the Prediction of Death for the WH Indicators (WH% and WHZ) for the WHO Growth Standards and the NCHS Standards

As an indicator for predicting mortality in the entire study population, NCHS reference using WHZ and WH% resulted in AUC values of 0.63 (CI 0.60–0.66) and 0.71 (CI 0.68–0.74), respectively. WHZ (WHO) and WH% (WHO) gave higher AUC values of 0.76 (CI 0.75–0.80) and 0.77 (CI 0.7–0.80), respectively. The AUCs of the WHO indicators were higher compared with any of the NCHS indicators (*p <* 0.001). The relationship between MUAC and risk of death was weaker (*p* = 0.05 compared to WHZ [NCHS], *p* < 0.001 compared to any of the three other WH indicators) with AUC = 0.63 (CI 0.60–0.67) (Table 3). Figure 2 presents the ROC curves for the WHO and NCHS criteria for WHZ and WH%.

**Table 3:**
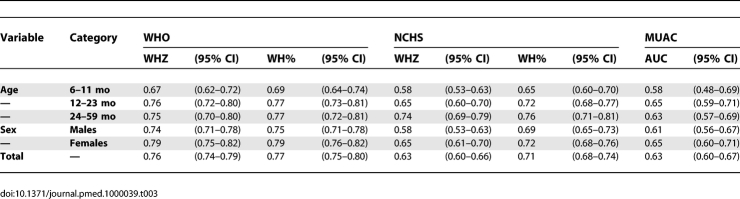
AUC for the WHO Growth Standards, the NCHS Reference, and the MUAC

The stratified analysis by age and sex also showed a better classification capacity with the WHO standards than the NCHS reference. The AUC was similar between girls and boys, but regarding age, all the criteria showed a lower level of discrimination for the youngest age group (Table 3).

### Cutoffs for WHO Standards and MUAC

**Figure 3:**
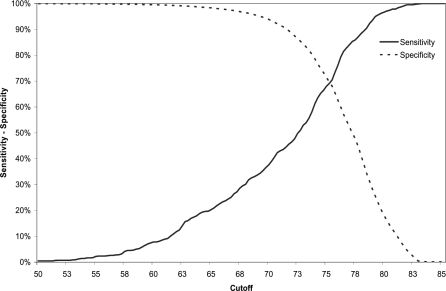
Sensitivity and Specificity for the Prediction of Death of the Cutoff for the WH% Using the WHO Growth Standards

**Figure 4:**
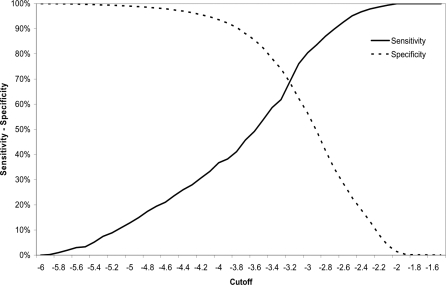
Sensitivity and Specificity for the Prediction of Death of the Cutoff for the WHZ using the WHO Growth Standards

The comparison between the different criteria showed the WHO standards to be superior in predicting deaths within the program than the NCHS reference. However, different cutoffs can be established resulting in different values of sensitivity and specificity. Figures 3 and 4 show the sensitivity and specificity for different cutoffs, and the distribution of children according to the WHO standards.

WHZ (WHO) varied from 0% to 100% of sensitivity and specificity in the range of values between −6 and −2 Z scores. The value used for defining severe malnutrition (−3 Z score) showed a sensitivity of 80.9% and a specificity of 55.0%. Decreasing the cutoff to −4.25, the sensitivity and specificity values were 30.0% and 96.0%. The higher specificity would mean a reduction in false positives and also in the number of children considered as severely malnourished. WH% (WHO) moved from 0% to 100% of sensitivity and specificity between the 55% and 85% of the median. The value used for defining severe malnutrition (<70% of the median) showed a sensitivity of 30.0% and a specificity of 96%. Table 4 displays cutoffs and specificities corresponding to different levels of sensitivities for the prediction of death.

**Table 4:**
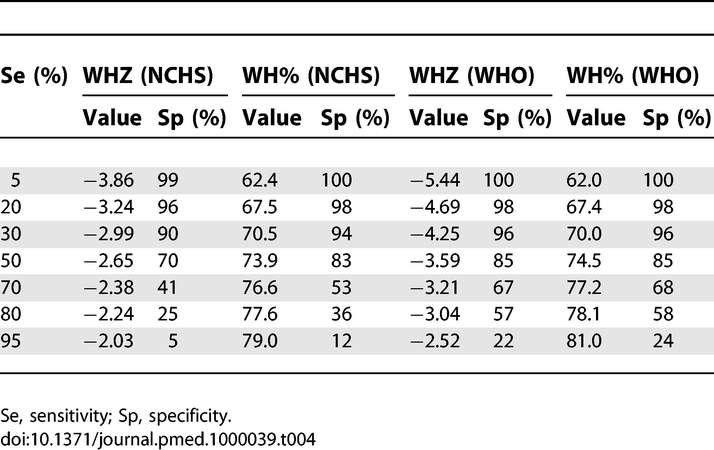
Correspondent Cutoffs for the WHO Growth Standards (WHO) and the NCHS Reference, Achieving the Same Levels of Sensitivity (Se) or Specificity (Sp) for the Prediction of Death

For the MUAC for all ages, the sensitivity obtained using the severe malnutrition cutoff of 110 mm was lower than 30%. The cutoff used to define moderate malnutrition, 125 mm, revealed different sensitivities for the three age groups investigated: 79.4% for 6–11 mo, 69.2% for 12–23 mo, and 60.5% for 24–59 mo. Similarly, if we define 70% as the minimum desired sensitivity, the cutoffs would be 122 mm, 125 mm, and 128 mm for the three age groups, respectively.

## Discussion

In a population of malnourished children admitted to an MSF nutritional program, the results suggest that WH using WHO standards was the best indicator to predict mortality under treatment with Z score and percentage of median providing nearly the same performance. Regarding the NCHS reference, the results are consistent with previous studies finding that WH% (NCHS) performed better than WHZ (NCHS) to predict mortality [9].

When stratifying by age classes, all the indicators performed more poorly in the youngest age group (Table 3). Children in this group are the most vulnerable and have the highest fatality rate. The variability in the causes of deaths is likely to be important, and the proportion of deaths related to other causes than malnutrition is higher among the youngest age group than among older children. This might explain the lower AUC to estimate the relation between nutritional status and mortality risk for any indicator. It is also possible that the youngest children responded more poorly to treatment.

Strengths and weaknesses exist among the different anthropometric indicators. For example, when focusing on even the highest AUC observed (AUC = 0.79 for WHZ [WHO] or WH% [WHO] for girls 6–59 mo) the sensitivity corresponding to a specificity of 80% is 40% and the specificity corresponding to a sensitivity of 80% is 35%. Thus, the choice of indicators must be evaluated in each specific context depending on what trade-off between sensitivity and specificity is tolerable. It is also important to emphasize that the choice of a cutoff depends on other factors besides sensitivity and specificity, for example, mortality risk without treatment, adverse events and risk of treatment, and response to treatment itself. This choice must take into account the means and objectives of the entire nutritional program.

Although the results of this study are informative, the results reported here were derived from a selected sample of the general population (i.e., children admitted to the MSF nutritional program). This sample selection has implications for the interpretation of results, which cannot be extrapolated to the general population (e.g., the cutoffs WH% [NCHS] = 80% or WHZ [NCHS] = 2.0 correspond to a sensitivity of 100% for the prediction of death, which would never occur in the whole population). Moreover, since all children in the database were included in a nutritional and medical program, it is impossible to predict how these children's clinical course would have evolved had they not been treated. A possible effect of this program may be to have lessened the observed performance of all the indicators, since we can suppose that children who would otherwise have died may have survived, which reduced the link between nutritional status on admission and mortality risk.

Rather than predicting death among admitted children, a far more important question would be to know who among all the malnourished children would benefit most from admission in the program in order to reduce mortality. Our results may help to answer this question, but do not bring sufficient evidence for extrapolation. Thus, using only our results to select children for admission to a nutritional program would not be appropriate. Also, it is important to point out that although all children in the program were diagnosed as malnourished, the cause of their death may be related to the interaction between malnutrition and other co-morbid conditions, such as malaria, or any other number of factors specific to the MSF program.

Several further limitations require note. The majority of children lost to follow-up were moderately malnourished at admission to outpatient treatment. These children are at low mortality risk and the most probable scenario is that the mother of these children, after a slight improvement of the nutritional status of the child, decided to leave the program because attendance at the outpatient treatment centers was too difficult in light of their daily activities. A further limitation could be evoked because of our choice to include defaulters in this study, since nonresponse to treatment is a negative outcome. However, nonresponse to treatment was not the principal objective of this study and we instead focused on probability of death. Excluding defaulters from the sample population would not qualitatively change the results (unpublished data). Finally, children with missing information on edema were assumed not to have bipedal edema. In a complementary analysis excluding these children, the results were similar and the rank of indicators remained unchanged.

It is also important to note that our database is constructed from program monitoring data, which have a different purpose than data collected for a clinical study. For example, the frequency of children aged exactly 12, 24, 36, or 48 mo was higher than what would be expected. This lack of accuracy may have direct consequences on our analyses, especially in the analyses stratified by age, but the similar results obtained in this analysis indicate a low impact of this potential bias. Obtaining accurate data on age is an ongoing problem. Ideally, comparison of the predictive value of different indicators for the whole age range for which an intervention is considered should be conducted and not simply comparing indicators for three age groups.

Another limitation relates to the admission criteria of the program. Children were included primarily due to the WH% (NCHS) <80% criteria, and not the MUAC <110 mm. Both the NCHS reference and the WHO standards present similar values of sensitivity for defining moderate malnutrition. Applying either of these criteria means similar populations would have been selected and therefore should not affect our results. On the contrary, the selection of children using the MUAC would have differed from the current sample. This fact may partly explain the unexpected weakness of MUAC to predict deaths, whereas it had previously been identified as a useful and important screening tool to rapidly assess nutritional status and risk of death of children, particularly in emergency settings [10–14]. A complementary hypothesis could suggest that the link between MUAC and deaths in previous studies may sometimes be due to additional factors (comorbidities including infections), which were mostly treated in this program.

If these results were confirmed by a study including children regardless of their nutritional status, this may have important implications for the use of MUAC as a diagnostic tool for malnutrition in therapeutic programs. MUAC changes with age and using it as tool in an emergency clearly serves a purpose as a sufficient and rapid tool, but in a stable nutritional program it is potentially limited in its use as a unique criterion. Several attempts have already been made more than 20 y ago [15–17] to correct MUAC in relation to height and age, which did not improve prediction (in practice, correcting MUAC for age or height leads to the selection of older children with a lower risk of dying and is not helpful). Further studies may nevertheless be needed to assess how the MUAC could be used in association with other anthropometric or clinical criteria to increase the ability to estimate the risk of death in such contexts.

In another study performed on the same database [18], we used multivariate logistic regression to assess which other factors were significantly related to mortality risk in a population of children with moderate malnutrition (defined with the WHZ [WHO] < −2 cutoff instead of the WH% [NCHS] cutoff), regardless of WH indicators. The results suggest that weight, height, presence of edema, and systematic use of a short list of clinical signs could be used to help identify children at highest risk of death in community-based programs in order to orientate the child towards either hospitalization or ambulatory care. The two studies address two different questions: this study examines differences in the diagnosis of malnutrition when comparing the WHO standards and the NCHS reference and in prognostic accuracy for predicting death at admission; the companion study examines clinical signs other than WH indicators as indicators of mortality risk [18]. However, the results of both studies are derived from a sample of admitted children, and cannot be considered as a screening tool to include children in nutritional programs.

These limitations point to the importance of future studies in a population more representative of all children being screened, examining both malnourished and healthy children when assessing the sensitivity and specificity of growth references. Because there were 11 outpatient and two inpatient centers, quality of care among these different centers could have varied, thereby confounding results. We also could have stratified the analysis into children admitted into inpatient or outpatient care, in essence, asking the question concerning how the indicators respond in two different populations. Grouping them together allowed us to not classify children a priori into two different groups, one with a higher expected mortality (inpatient) than the other.

Overall, this analysis of anthropometric data from over 60,000 children in a large-scale nutritional intervention program in Niger suggests that the new WHO growth standards are more accurate indicators for mortality risk in malnourished under-5-y populations during their stay in a nutritional program, compared with the older NCHS reference. This improved accuracy appears to hold whether using Z scores or percentage of the median to measure WH. Sensitivity and specificity in relation to survival should be reexamined taking a more representative sample of children, in order to assess who would benefit most from the nutritional program in order to reduce mortality.

## Abbreviations

AUC: area under curve
CI: 95% confidence interval
MSF: Médecins Sans Frontières
NCHS: National Center for Health Statistics
ROC: receiver operating characteristic
RUTF: ready to use therapeutic food
WH: weight-for-height
WH%: weight-for-height percentage of the median
WHO: World Health Organization
WHZ: weight-for-height Z score

## References

[pmed-1000039-b001] WHO Multicentre Growth Reference Study Group (2006). WHO Child Growth Standards: Length/height-for-age, weight-for-age, weight-for-length, weight-for-height and body mass index-for-age: Methods and development.

[pmed-1000039-b002] de Onis M, Onyango AW, Borghi E, Garza C, Yang H (2006). Comparison of the World Health Organization (WHO) Child Growth Standards and the National Center for Health Statistics/WHO international growth reference: implications for child health programmes. Public Health Nutr.

[pmed-1000039-b003] Seal A, Kerac M (2007). Operational implications of using 2006 World Health Organization growth standards in nutrition programmes: secondary data analysis. BMJ.

[pmed-1000039-b004] World Health Organization. Country Profiles: Niger.

[pmed-1000039-b005] National Institute of Statistics (INS), Niger and Macro International Inc. (2007). Demographic Health Survey/Multiple Indicator Cluster Survey 2006 (DHS/MICS III) Preliminary Report.

[pmed-1000039-b006] National Institute of Statistics (INS), Niger, United Nations Children's Fund and the World Food Programme (2007). Survey of mortality and nutrition among children aged 6–59 mo in Niger, October–November 2006.

[pmed-1000039-b007] World Health Organization; World Food Programme; United Nations. Standing Committee on Nutrition; UNICEF (2007). Community-based management of severe acute malnutrition. A joint statement by the World Health Organization, the World Food Programme, the United Nations System Standing Committee on Nutrition and the United Nations Children's Fund.

[pmed-1000039-b008] DeLong ER, DeLong DM, Clarke-Pearson DL (1988). Comparing the areas under two or more correlated receiver operating characteristic curves: a nonparametric approach. Biometrics.

[pmed-1000039-b009] Prudhon C, Briend A, Laurier D, Golden MH, Mary JY (1996). Comparison of weight- and height-based indices for assessing the risk of death in severely malnourished children. Am J Epidemiol.

[pmed-1000039-b010] Briend A, Dykewicz C, Graven K.Show (1986). Usefulness of nutritional indices and classifications in predicting death of malnourished children. BMJ.

[pmed-1000039-b011] Myatt M, Khara T, Collins S (2006). A review of methods to detect cases of severely malnourished children in the community for their admission into community-based therapeutic care programs. Food Nutr Bull.

[pmed-1000039-b012] Bern C, Nathanail L (1995). Is mid-upper-arm circumference a useful tool for screening in emergency settings. Lancet.

[pmed-1000039-b013] Mei Z, Grummer-Strawn LM, de Onis M, Yip R (1997). The development of a MUAC-for-height reference, including a comparison to other nutritional status screening indicators. Bull World Health Organ.

[pmed-1000039-b014] de Onis M, Yip R, Mei Z (1997). The development of MUAC-for-age reference data recommended by a WHO Expert Committee. Bull World Health Organ.

[pmed-1000039-b015] Briend A, Zimicki S (1986). Validation of arm circumference as an indicator of risk of death in one to four year old children. Nutr Res.

[pmed-1000039-b016] Briend A, Garenne M, Maire B, Fontaine O, Dieng K (1989). Nutritional status, age and survival: the muscle mass hypothesis. Eur J Clin Nutr.

[pmed-1000039-b017] Briend A, Wojtyniak B, Rowland MGM (1987). Arm circumference and other risk factors in children at high risk of death in rural Bangladesh. Lancet.

[pmed-1000039-b018] Lapidus N, Minetti A, Djibo A, Guerin PJ, Gaboulaud V (2009). Risk factors for mortality among children admitted to a large-scale nutritional program in Niger, 2006. PLoS One.

